# Treatment Adherence and Its Associated Factors in Patients with Type 2 Diabetes: Results from the Rio de Janeiro Type 2 Diabetes Cohort Study

**DOI:** 10.1155/2018/8970196

**Published:** 2018-11-27

**Authors:** Fernanda S. Marinho, Camila B. M. Moram, Priscila C. Rodrigues, Nathalie C. Leite, Gil F. Salles, Claudia R. L. Cardoso

**Affiliations:** ^1^Department of Occupational Therapy, University Hospital Clementino Fraga Filho, School of Medicine, Universidade Federal do Rio de Janeiro, Brazil; ^2^Department of Internal Medicine, University Hospital Clementino Fraga Filho, School of Medicine, Universidade Federal do Rio de Janeiro, Brazil

## Abstract

**Objectives:**

To investigate treatment adherence in patients with type 2 diabetes and to evaluate its associated factors.

**Methods:**

The Summary of Diabetes Self-Care Activities (SDSCA) questionnaire was used to assess treatment adherence. Good adherence was defined as ≥5 days a week in each SDSCA item. Pain, emotional, and physical domains of the SF-36 quality of life questionnaire and the Canadian Occupational Performance Measure (COPM) were also evaluated. Multivariable logistic regressions explored the independent correlates of good general adherence and of specific items of the SDSCA (diet, exercise, and medications).

**Results:**

Good adherence was 93.5% for medication use, 59.3% for foot care, 56.1% for blood glucose monitoring, 29.2% for diet, and 22.5% for exercise. Patients with general good adherence had lower BMI, better serum lipid profile, higher values of functional capacity, emotional and pain domains of SF-36, better occupational performance, and lower prevalence of pain or limitation in the upper and lower limbs than patients with worse adherence. The variables associated with good adherence were younger age, lower BMI, presence of macrovascular complications, better occupational performance and emotional domain of SF-36, and higher HDL cholesterol levels. The presence of pain/limitation in the upper limbs was associated with worse adherence. Good medication adherence was associated with longer diabetes duration, lower BMI, and lower HbA_1c_ levels. Higher values of pain and emotional domains of the SF-36 and lower BMI were related to better exercise and diet adherence, while the presence of peripheral neuropathy and joint pain/limitation were associated with worse exercise adherence.

**Conclusions:**

Emotional and physical performances are important determinants of good diabetic treatment adherence. Good adherence has beneficial impact on BMI, lipid, and glycemic control.

## 1. Introduction

Type 2 diabetes prevalence is progressively growing worldwide due to increases in population ageing and obesity. Around 451 million adults are estimated to have diabetes, most of them living in low- and middle-income countries. If these trends continue, by year 2045, 693 million people will have diabetes [1]. Chronic diabetic complications lead to increased morbidity and precocious mortality due to inadequately controlled diabetes, causing an important burden to individuals, families, society, and health care systems worldwide [2].

Subjects with diabetes need to perform self-care activities to prevent short- and long-term complications related to inadequate disease control and to improve their quality of life. Self-care is considered the keystone of diabetes treatment. Hence, to evaluate the proper adherence to diabetes self-care is fundamental to identify and understand the trouble areas in diabetes management. This may help to improve diabetes control and to decrease the burden of complications. Among the questionnaires used to evaluate diabetes self-care, the most commonly employed is the Summary of Diabetes Self-Care Activities (SDSCA). This instrument has been evaluated in many studies and demonstrated satisfactory psychometric properties [3].

It has been suggested that psychosocial factors, such as depression and emotional stress [4–6], as well as the development of chronic degenerative complications, particularly peripheral neuropathy, are associated with worsening of general health condition [7]. Furthermore, the presence of functional disability, defined as difficulty in executing daily life activities and the tasks necessary for the independent functioning in daily life instrumental activities, may also impact on diabetes self-care [8, 9]. Functional disability seems to be more frequent in older adults with diabetes than in those without diabetes [9–11]. The most commonly used strategy to evaluate disabilities is based on self-reported levels of difficulty in performing mobility tasks, in instrumental activities of daily living and in basic activities of daily living. The Canadian Occupational Performance Measure (COPM) is an outcome measure designed to aid patients to recognize, prioritize, and evaluate the most important disabilities they find in their occupational performance [12]. All these factors, in conjunction, may lead to a decrease in adherence to diabetes self-care daily activities. Hence, to identify the factors associated with better or worse adherence to diabetes self-care is potentially important to achieve future better outcomes in type 2 diabetes management.

Therefore, we intended to investigate the self-care behavior of middle-aged to elderly type 2 diabetes individuals, using the SDSCA questionnaire, and the factors associated with good adherence to self-care recommendations. In particular, we investigate the associations with the presence of chronic degenerative complications, with emotional, physical, and pain domains of life quality evaluated by the Medical Outcomes Study 36-Item Short-Form Health Survey (SF-36) questionnaire, with the profile of disabilities evaluated by the COPM and, additionally, with parameters of diabetes control.

## 2. Methods

This was a cross-sectional study nested within the Rio de Janeiro Type 2 Diabetes Cohort Study, with 476 type 2 diabetic patients in regular attendance in the outpatient clinic of a tertiary care university hospital. The specific exclusion criteria of this study were patients with difficulties to understand the questionnaire due to cognitive problems and patients who did not agree or could not participate in the study. All participants gave written informed consent, and the local Ethics Committee had previously approved the study protocol. The characteristics of this cohort, the baseline procedures, and the diagnostic definitions have been detailed elsewhere [13–15]. All patients were submitted to a standard protocol at a study entry that included a complete clinical examination and laboratory evaluation [13–15]. Pain or joint limitations, which hindered the performance of some daily activities, were investigated by a standard questionnaire that included inquiries on the spine and lower and upper limbs. The SF-36 questionnaire was used to evaluate pain and emotional and physical functional domains of life quality, which has been translated and validated in Brazil [16]. Higher values on SF-36 mean better quality of life in these domains. Subjects were also interviewed to identify the activities that they presented greater difficulty to perform by the Canadian Occupational Performance Measure (COPM), as previously described [17]. Higher values in the COPM mean better occupational performance.

### 2.1. Summary of Diabetes Self-Care Activities (SDSCA) Questionnaire

The revised Summary of Diabetes Self-Care Activities (SDSCA) questionnaire was employed, translated to Portuguese, and previously validated to the Brazilian population [18]. The evaluation was performed from February 2012 to February 2013, by individual interviews conducted by a single experienced occupational therapist. The SDSCA questionnaire evaluates the performance of activities by the patients and their compliance with prescription and other health care professional recommendations. It assesses aspects of the treatment regimen of diabetes over the previous seven days, grouped into six self-care dimensions: diet care (general and specific), physical activity, use of medication, blood glucose monitoring (evaluated only on the 311 patients using insulin), foot care, and smoking. Response options range from 0 to 7 to match the number of days of the week. The higher the number of days per week performing that activity, the higher the level of adherence to this recommendation. The questionnaire also evaluates the average number of cigarettes smoked per day. Patients were considered having general good adherence to diabetic treatment if they were not current smokers and reported at least 5 days per week of compliance to diet, exercise, foot care, and medication use. However, for the questions of eating high fat foods and meals including sweets (specific diet), it was considered good adherence ≤ 2 days per week. The 5-day criterion was also used for the other separate item of the SDSCA questionnaire.

### 2.2. Statistical Analysis

Continuous variables were described as the means and SDs when normally distributed and as the medians and interquartile range when asymmetrically distributed. An unpaired *t*-test or Mann-Whitney tests and chi-squared tests, where adequate, were used to compare variables between patients according to general adherence to diabetes treatment. The covariates independently associated with general good adherence and separated for adherence to diet, physical activity, and medication use were assessed by multivariate logistic regressions using a backward stepwise selection procedure, where a *p* value < 0.10 was the criterion to remain into the final models. The candidate variables to enter the models, based on biological plausibility, were age, sex, BMI, diabetes duration, SF-36 pain, physical activity and emotional domains, pain or joint limitations on the spine and lower and upper limbs, COPM score (occupational performance), presence of each macrovascular and microvascular complications, clinic blood pressure levels, glycated hemoglobin (HbA_1c_), and serum lipid levels (LDL- and HDL cholesterol and triglycerides). Age and sex were forced into all models, regardless of their significance. When the COPM score was entered as a candidate variable into the multivariate analysis, the SF-36 physical activity domain was not included. Similarly, when pain or joint limitations on the spine and lower and upper limbs were entered into the multivariate analysis, the SF-36 pain domain was not included. Results were presented as the odds ratios with their respective 95% confidence intervals. All statistics were performed with the SPSS statistical package version 19.0 (SPSS Inc., Chicago, IL, USA), and a two-tailed *p* value < 0.05 was regarded significant.

## 3. Results

### 3.1. The Summary of Diabetes Self-Care Activities

**Figure 1:**
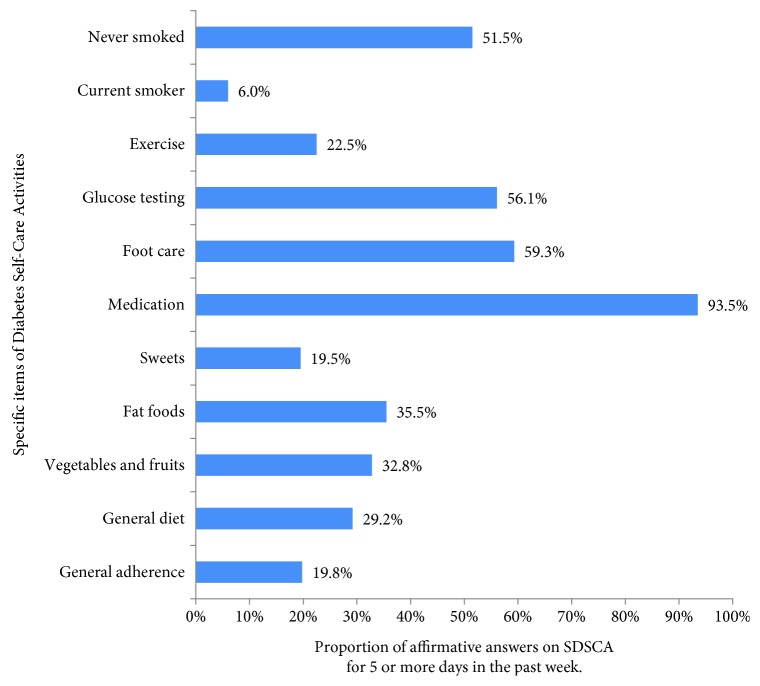
Summary of Diabetes Self-Care Activities and percentage of affirmative answers to SDSCA of 5 days or more, except for sweets and fat food that were considered affirmative answers to two days or less.

Table 1 presents the results of specific items of the SDSCA questionnaire, and Figure 1 outlines the proportion of patients considered adherent (5 or more days in the last week) for each SDSCA item. In general, patients had a poor adherence to diet and exercise, a moderate adherence to regular blood sugar assessments and foot care, and a good adherence to medications and not smoking. Overall, only 20% of the patients were considered with good general adherence to diabetes treatment.

### 3.2. Characteristics of All Patients and according to General Adherence

Table 2 presents data of all patients and of those considered overall adherent (≥5 days a week) and nonadherent (<5 days a week) by the self-reported SDSCA questionnaire. Patients with better adherence had a lower BMI and a better serum lipid profile, particularly higher HDL cholesterol and lower triglycerides levels, than nonadherent patients. They also had better physical, functional, emotional, and pain domains of the SF-36 questionnaire; better occupational performance evaluated by CPOM; and lower prevalences of limitation or pain in the upper and lower limbs than those patients with worse general adherence.

### 3.3. Variables Associated with General Adherence and with Specific Items of Diet, Exercise, and Medication Use of the SDSCA Questionnaire

Results of the multivariate logistic regression analyses of the independent correlates of optimal general adherence and of the specific correlates of diet, exercise, and medication adherence are presented in Table 3. Younger age, lower BMI, higher HDL cholesterol levels, and the presence of macrovascular complications were related to better general adherence. Higher values of the SF-36 emotional domain and better occupational performance were also associated with better general adherence, while the presence of limitation/pain in the upper limbs was associated with worse general adherence.

Good adherence to diet was associated with higher values of HDL cholesterol, better occupational performance measured by CPOM, and better pain domain of the SF-36 questionnaire, while the presence of limitation/pain in the upper limbs was associated with worse adherence to diet. Regarding adherence to regular exercise, better pain and emotional domains on the SF-36 questionnaire, lower BMI, and better occupational performance were independently associated with good adherence, while the presence of diabetic peripheral neuropathy and pain/limitation in the spine were associated with poor adherence to exercise. Better self-reported adherence to medication use was independently associated with lower HbA_1c_ levels, longer diabetes duration, and lower BMI.

## 4. Discussion

This study has three main findings. First, it demonstrated that adherence to self-care recommendations, evaluated by the SDSCA questionnaire, in middle-aged to elderly type 2 diabetes was good (>90%) only for medication use and nonsmoking. It was moderate for foot care (59%) and for blood glucose monitoring (56%) and was particularly poor (<30%) for diet and physical activity. Second, and the most important, better general adherence was associated with younger age, lower BMI, better occupational performance (reflected by higher values of the COPM, which is a measure of self-reported disabilities), better emotional domain of the SF-36 quality of life questionnaire, and higher values of HDL cholesterol. The presence of limitation/pain in the upper limbs was associated with worse general adherence. Third, good self-reported adherence to medication use was associated with better glycemic control, good adherence to diet was associated with better serum lipid profile, and a lower BMI was associated with almost all items of self-care activities, suggesting the importance of treatment adherence to better overall diabetes control. Furthermore, better scores of pain and emotional domains of the SF-36 questionnaire, better occupational performance, and absence of joint pain/limitation and of diabetic peripheral neuropathy were also associated with good adherence to diet and physical activity, reflecting the importance of physical and emotional aspects to better treatment adherence. These findings suggest that future studies with interventions to improve functional disabilities, especially by reducing pain in the upper limbs and spine and preventing peripheral neuropathy progression, and emotional support with treatment of depression/anxiety may help to improve overall adherence to diabetes treatment with consequent impact on better outcomes.

In this study, diabetes medication adherence was associated with lower HbA_1c_ levels. The relation between medication adherence and glucose control is still subject to debate, in which most studies showed significant associations between self-reported medication adherence and HbA_1c_, using different instruments [19–22], while others did not demonstrate associations between self-reported adherence and HbA_1c_ [23]. Nonetheless, associations with other measures of adherence and HbA_1c_ may be modest, or not present, because glycemic control is dependent of various factors, besides medication adherence, including diet and exercise, grade of insulin deficit, and adequacy of medication in use [24, 25]. Further, a meta-analysis [26] in type 1 diabetes investigating the relationship between treatment adherence and HbA_1c_ showed that adherence explained a small part of HbA_1c_ variation (<8%). It also demonstrated no difference in the strength of associations in studies that used self-reported or objectively monitored medication adherence [26]. Likewise, a study in type 2 diabetes assessing relations between medication adherence and HbA_1c_ demonstrated that adherence explained only 4% of baseline HbA_1c_ and 1.7% of HbA_1c_ change [27]. So validating self-reported adherence by using only clinical endpoints such as HbA_1c_ levels may lead to less precise inferences, due to rather modest influence of adherence on this outcome [24]. Additionally, methods for assessing medication adherence and persistence were highly variable and all meta-analyses had a high degree of heterogeneity [28]. Besides, younger age and longer diabetes duration were also independently associated with better medication adherence. These findings are in agreement, in part, with a large report [29] of diabetic patients treated with noninsulin medications where longer disease duration was associated with better adherence. However, different from our study, older age and male gender were also related to better adherence [29].

Depression has been frequently associated with self-reported worse treatment adherence in patients with diabetes [4, 30]. Similarly, lower scores on the emotional domain of the SF-36 questionnaire, which combines depressive and anxiety complains, were associated with worse general adherence in the present study. Additionally, we observed that pain/limitation in the upper limbs was independently associated with worse general adherence, which is a relevant finding. Although musculoskeletal disorders are sometimes unique or more prevalent in people with diabetes, conditions of the upper limb causing pain, discomfort, and limited movements have in general been under-diagnosed and poorly treated, in comparison to the other complications of diabetes [31]. Injury to vessels and nerves, protein glycosylation, and augmented collagen in the skin and musculoskeletal connective tissues are some factors that possibly may contribute to the development of musculoskeletal disorders in diabetic patients [32]. They may lead not only to important disabilities and poor quality of life in diabetic patients but also to poor treatment adherence and worse outcomes.

Not unexpectedly, the presence of peripheral neuropathy was associated with worse physical activity adherence and higher values of pain and emotional domains of SF-36 were related to better adherence. Previous studies reported relationships between the presence of neuropathy and depression [33, 34]. However, the association between the presence of depression and peripheral neuropathy and reduced treatment adherence is less evidenced in diabetic patients [35].

There are limitations in the present study that need to be acknowledged. First, its cross-sectional design allows no speculations about causality, but only associations between adherence and the factors. More importantly, we investigated self-reported treatment adherence, which may result in biased data. However, even objective measurements of adherence, which have been demonstrated to be comparable to self-reported adherence in their associations with glycemic control, [25] have also their own related measurement error [24]. Furthermore, objective measurements of treatment adherence frequently are not practical in clinical settings and are not able to help caregivers to improve treatment adherence of their patients [24]. Another aspect that restricts performing research on treatment adherence is the absence of a standard definition, which limits the comparability of studies and increases the risk of selective reporting [24]. The team of health care providers is compounded of three medical doctors, two nurses, two occupational therapists, and a nutritionist; although the group is homogeneous regarding commitment and support to patients, we did not evaluate if there were differences in the adherence among them, considering that these factors may have a relation with adherence. Finally, this study was conducted in a tertiary care hospital with a patient population of predominantly middle-aged to elderly individuals; thus, our findings may not be generalizable to younger diabetic patients and to patients followed at primary care centers. On the other hand, we included a relatively large number of type 2 diabetic patients, and its main strength is that our well-documented cohort was allowed to perform a comprehensive analysis of possible factors associated with adherence.

In conclusion, this study demonstrated that general adherence to recommended self-care activities in middle-aged to elderly patients with type 2 diabetes was poor, particularly because of poor adherence to diet and exercise, that several emotional and physical factors seemed to affect adherence, and that good adherence was associated with better diabetes control. This study suggests that patient-centered interventions addressing improvements in different areas of performance, including pain management and emotional support, may enable greater patient independence and autonomy and may also improve treatment adherence. Whether such interventions will have impact on better clinical outcomes in patients with type 2 diabetes shall be the focus of future studies.

## Figures and Tables

**Table 1:** Results of the Summary of Diabetes Self-Care Activities (SDSCA) questionnaire in 476 type 2 diabetic patients.

Self-care activities	Number of days^∗^
General diet questions	
Follow a healthful eating plan in the last week	0 (0–6)
Follow an eating plan (on average per week, over the past month)	0 (0–6)
Specific diet questions	
Eat five or more servings of fruits and vegetables in the last week	0 (0–7)
Eat high-fat foods in the last week	4 (2–7)
Eat sweets in the last week	1 (0–2)
Exercise	
Participate in at least 30 minutes of physical activity, in the last week	7 (3–7)
Participate in a specific exercise session in the last week	0 (0–2)
Blood sugar testing (patients using insulin = 311)	
Test blood sugar in the last week	2 (0–7)
Test blood sugar according to the number of times recommended by your health care provider in the last week	5.5 (0–7)
Foot care	
Check feet in the last week	7 (1–7)
Inspect the inside of shoes in the last week	7 (0–7)
Dry between toes after washing, in the last week	7 (7–7)
Medications	
Take recommended diabetic medications in the last week	7 (7–7)
Take recommended insulin injections in the last week	7 (7–7)
Take recommended number of diabetes pills in the last week	7 (7–7)
Smoking	
Patients who smoked during the past 7 days (%)	6.1
Number of cigarettes smoked in a day	13 (5–15)
Never smokers (%)	51.5
Past smokers (%)	42.4

^∗^Values are the median number of days and interquartile range, except for smoking status, which are proportions and median number of cigarettes (for current smokers).

**Table 2:** Characteristics of all patients and according to general adherence (5 or more days per week in all items of the SDSCA questionnaire, except in exercise).

Characteristics	All patients (*n* = 476)	Adherent patients (*n* = 94)	Nonadherent patients (*n* = 382)	*p* value
Age (years)	65 (10.7)	64 (11.7)	65 (10.5)	0.29
Diabetes duration (years)	8 (3–15)	8 (3–15)	8 (3–15)	0.94
Gender, female (%)	63.9	63.6	64.9	0.82
Schooling years, ≥8 years (%)	25.5	24.9	27.5	0.59
Social security situation (% of retirement)	38	43.6	36.6	0.69
Marital status (% of married)	64.1	70.1	62.6	0.48
Body mass index (kg/m^2^)	29.8 (4.7)	28.7 (4.1)	30.1 (4.8)	0.005
Diabetes treatment (%)				
Metformin	86.8	86.6	86.9	0.95
Sulfonylureas	20.8	21.5	20.6	0.86
Insulin	68.4	67.7	68.5	0.89
Chronic diabetic complications (%)				
Retinopathy	31.0	34.8	30.1	0.38
Nephropathy	28.2	28.0	28.2	0.96
Peripheral neuropathy	27.4	24.5	28.2	0.47
Cerebrovascular disease	6.5	7.4	6.3	0.68
Coronary artery disease	14.7	16.0	14.4	0.70
Peripheral arterial disease	13.5	12.6	17.0	0.26
Arterial hypertension (%)	85.3	81.9	86.1	0.30
SBP (mmHg)	139 (18)	139 (20)	138 (18)	0.81
DBP (mmHg)	76 (11)	75 (11)	76 (11)	0.34
Dyslipidemia (%)	87.6	85.1	88.2	0.41
Laboratory variables				
HbA_1c_ (%) (mmol/mol)	7.9 (1.6)	7.9 (1.6)	7.9 (1.7)	0.98
	63 (15.6)	63 (15.6)	63 (15.7)	
LDL cholesterol (mmol/L)	2.46 (0.78)	2.45 (0.69)	2.46 (0.81)	0.92
HDL cholesterol (mmol/L)	1.19 (0.36)	1.27 (0.36)	1.17 (0.35)	0.007
Triacylglycerol (mmol/L)	1.46 (1.07–2.04)	1.29 (0.97–1.73)	1.50 (1.11–2.13)	0.003
Domains of quality of life (SF-36)				
Functional capacity	49 (31.5)	56 (32.3)	47 (31.1)	0.011
Emotional	58 (45.6)	68 (43.3)	55 (46.0)	0.014
Pain	51 (30.3)	56 (30.8)	49 (30.1)	0.041
Occupational performance (COPM)	4.6 (1.9)	5.2 (2.0)	4.4 (1.9)	0.001
Satisfaction in the performance of activities (COPM)	4.3 (2.3)	4.8 (2.5)	4.2 (2.2)	0.035
Limitations/pain (%)				
In the upper limb	52.3	37.2	56.0	0.001
In the lower limb	60.3	47.9	63.4	0.006
In the spine	54.8	54.3	55.0	0.90

Values are the proportions, means (standard deviations), or medians (interquartile range). Abbreviations: SBP, systolic blood pressure; DBP, diastolic blood pressure; HbA_1c_, glycated hemoglobin; HDL, high-density lipoprotein; LDL, low-density lipoprotein; COPM, Canadian Occupational Performance Measure.

**Table 3:** Results of multiple logistic regressions for variables associated with general adherence and, for specific items of diet, exercise and medication adherence of the SDSCA questionnaire.

Dependent variable/independent covariates	OR	95% CI	*p* value
General adherence^∗^			
HDL cholesterol (10 mg/dL increase)	1.25	1.06–1.47	0.008
SF-36 emotional domain (each 10-point increase)	1.07	1.01–1.12	0.021
BMI (1 kg/m^2^ increase)	0.94	0.89–0.99	0.021
Age (1 year increase)	0.98	0.96–0.998	0.033
Presence of macrovascular complications (yes/no)	1.62	0.96–2.37	0.073
General adherence^∗∗^			
Pain/limitation in the upper limbs (yes/no)	0.49	0.30–0.81	0.006
HDL cholesterol (10 mg/dL increase)	1.25	1.06–1.47	0.008
COPM (each 1-point increase)	1.18	1.04–1.35	0.012
Age (1-year increase)	0.97	0.95–0.99	0.014
SF-36 emotional domain (each 10-point increase)	1.07	1.01–1.12	0.021
Presence of macrovascular complications (yes/no)	1.88	1.09–3.23	0.022
BMI (1 kg/m^2^ increase)	0.96	0.91–1.01	0.098
General diet^∗^			
HDL cholesterol (10 mg/dL increase)	1.19	1.02–1.37	0.022
SF-36 pain domain (each 10-point increase)	1.08	1.01–1.16	0.033
SF-36 emotional domain (each 10-point increase)	1.04	0.99–1.09	0.076
General diet^∗∗^			
COPM (each 1-point increase)	1.22	1.09–1.36	0.001
Pain/limitation in the upper limbs (yes/no)	0.59	0.38–0.90	0.015
HDL cholesterol (10 mg/dL increase)	1.19	1.02–1.38	0.025
Exercise^∗^			
SF-36 pain domain (each 10-point increase)	1.13	1.04–1.21	0.002
SF-36 emotional domain (each 10-point increase)	1.09	1.03–1.15	0.003
BMI (1 kg/m^2^ increase)	0.94	0.89–0.99	0.011
Presence of peripheral neuropathy (yes/no)	0.55	0.31–0.95	0.032
Exercise^∗∗^			
SF-36 emotional domain (each 10-point increase)	1.08	1.02–1.14	0.009
BMI (1 kg/m^2^ increase)	0.94	0.90–0.99	0.029
Presence of peripheral neuropathy (yes/no)	0.55	0.31–0.95	0.032
Pain/limitation in the spine (yes/no)	0.61	0.39–0.98	0.039
COPM (each 1-point increase)	1.12	0.99–1.27	0.066
Female gender	1.53	0.95–2.45	0.082
Medication^†^			
HbA_1c_ (1% increase)	0.73	0.62–0.86	<0.001
BMI (1 kg/m^2^ increase)	0.92	0.86–0.99	0.022
Diabetes duration (1-year increase)	1.04	0.99–1.09	0.059

^∗^The candidate variables to enter these models were BMI, diabetes duration, SF-36 pain, physical activity and emotional domains, presence of macrovascular and microvascular complications, blood pressure, glycated hemoglobin, and LDL and HDL cholesterol. ^∗∗^Pain or joint limitation on the spine and lower and upper limbs and COPM entered the model instead of SF-36 pain and physical domain items. ^†^Both models were identical. Age and gender were forced into all models. Abbreviations: BMI, body mass index; COPM, Canadian Occupational Performance Measure.

## Data Availability

The Rio de Janeiro Type 2 Diabetes Cohort Study is an ongoing study, and its dataset is not publicly available due to individual privacy of the participants. However, it may be available from the corresponding author on reasonable request.
